# Management and outcome of Bacille Calmette-Guérin vaccine adverse reactions

**DOI:** 10.1016/j.vaccine.2015.07.103

**Published:** 2015-10-05

**Authors:** Aishwarya Venkataraman, Michael Yusuff, Susan Liebeschuetz, Anna Riddell, Andrew J. Prendergast

**Affiliations:** a Barts Health NHS Trust, London, UK; b Centre for Paediatrics, Blizard Institute, Queen Mary University of London, London, UK

**Keywords:** BCG vaccine, Lymphadenitis, Complications, Treatment, Outcome

## Abstract

**Background:**

Bacille Calmette-Guérin (BCG) vaccine is one of the most widely used vaccines globally. Management of local BCG complications (injection site reactions and suppurative or non-suppurative lymphadenitis) varies between clinicians, and the optimal approach remains uncertain.

**Aim:**

To determine the clinical features, management and outcome of BCG complications at two large acute hospitals in London, United Kingdom.

**Methods:**

All children presenting with complications of BCG vaccination between January 2008 and December 2013 were included in this observational study. Medical and electronic laboratory records were reviewed to determine clinical features, treatment and outcome.

**Results:**

Sixty children presented with adverse reactions. Two-thirds (65%) presented with BCG lymphadenitis, one-third (30%) presented with injection site complications and two children (3%) presented with both injection site reaction and lymphadenitis; only one child (2%) had disseminated BCG disease. The majority (88%) of children with injection site reactions were managed conservatively; overall, 95% showed complete resolution within 6 months. Among children with lymphadenitis, 46% were managed conservatively, whilst 54% had anti-tuberculous therapy and/or a procedure (aspiration mostly, or surgery); complete resolution was seen in 59% of cases.

**Conclusions:**

Injection site reactions and non-suppurative lymphadenitis were generally managed conservatively, with good outcomes. There was more variation in management and outcome of suppurative lymphadenitis and the optimal approach remains uncertain.

## Highlights

• BCG vaccine is the one of the most widely used vaccines globally.
• Local complications are commonly seen but approach to management varies.
• Injection site reactions and non-suppurative lymphadenitis can be managed conservatively.
• Aspiration may be beneficial in suppurative lymphadenitis.
• Antimicrobials and surgery are rarely required except for non-resolving cases.

## 1 Introduction

The Bacille Calmette-Guérin (BCG) vaccine is a live attenuated strain of *Mycobacterium bovis*, first used in humans in 1921. Overall, BCG vaccine reduces the risk of pulmonary and extra-pulmonary tuberculosis (TB) by approximately 50% [1–3], but it has 64% efficacy against TB meningitis and 78% against disseminated TB disease [1,2,4]. BCG vaccine also provides some protection against leprosy and non-tuberculous mycobacterial infections [5]. In addition, it has been used in the treatment of superficial carcinoma of the bladder [6,7].

A typical reaction following BCG vaccination is a red indurated area at the injection site, which may subsequently ulcerate, then form a crust, which falls off after about 6 weeks leaving a small scar. Axillary lymphadenopathy (<1 cm) is also a normal response to BCG vaccination [4,8]. Adverse reactions to BCG vaccine are reported in 1–10% of vaccinees, but are likely to be substantially underreported [9,10]. Adverse reactions are usually seen within the first 6 months of vaccination but can present beyond 12 months [10,11]. There appears to be an association between adverse reactions and the strain of BCG administered [10,12–15]. Worldwide, the most commonly used vaccine strains currently are Statens Serum Institute (SSI) Copenhagen strain 1331 (Danish 1331), Tokyo 172-1 and Russian BCG-I [15]. In the United Kingdom, Danish 1331 has been in use since 2004. Depending on the immunogenicity generated in animal models, strains have been termed strong (e.g. Danish 1331) or weak (e.g. Tokyo 172) [12,15], with strong strains associated with more adverse reactions [12,13,15].

BCG complications can cause significant morbidity in children and anxiety in their parents. Local adverse reactions include regional suppurative and non-suppurative lymphadenitis, injection site abscesses, persistent injection site reactions, ulceration and uncommonly keloid reactions [3,10,16]. Systemic adverse reactions are rare and include osteomyelitis/osteitis and disseminated BCG disease [3,10]. Systemic dissemination is of particular risk in children with primary immune deficiencies or human immunodeficiency virus (HIV) infection [17]. Local and regional adverse reactions occur most frequently and the majority are self-limiting [4,17]. However, many clinicians advocate treatment, including use of antimicrobials, needle aspiration and/or surgery [4,11,17] and practice is likely to vary substantially across sites.

Here we describe our experience of BCG vaccine-associated adverse reactions in children referred to two large hospitals in London, United Kingdom.

## 2 Methods

### 2.1 Study subjects

We included all children presenting between January 2008 and December 2013 with complications associated with BCG vaccination to two large acute hospitals (Royal London Hospital and Newham University Hospital) in east London, UK. In the UK, BCG vaccination is usually given in the community, once the baby is discharged home after birth. Children with BCG complications are mostly seen by general practitioners initially; those needing specialist review are referred to hospital. We identified children through clinic lists (general paediatrics, infectious diseases and TB clinics) and referral letters (from general practitioners or other specialists) and reviewed their medical records and electronic laboratory data. Our study includes children who were initially referred to other specialties (general surgery and orthopaedic surgery) but were subsequently referred to a paediatric clinic for review. Data on the following variables were collected onto a proforma: baseline demographics, age at presenting to hospital with adverse reactions, details of vaccination procedure (if available), type of complication, microbiological investigations undertaken, management approach and outcome.

Formation of a small scar after vaccination was considered a normal reaction. Complications were defined as local (injection site reaction, isolated lymphadenitis or both) or systemic (osteomyelitis/osteitis and disseminated BCG disease). Lymphadenopathy >1 cm in size and presence of an abscess/ulcer at the injection site beyond 12 weeks were considered to be complications. Treatment given was divided into conservative (watch and wait), medical therapy (antimicrobials), needle aspiration or surgery. Outcome was defined as either complete resolution (a small scar at the injection site within 6 months of presentation) or healing with complications (sinus, ulcer, keloid, persistent lymphadenitis beyond 6 months or significant scarring requiring referral to a plastic surgeon or dermatologist), as judged by the treating clinician. Children who were seen in the clinic with a normal oozing injection site (mostly due to parental anxiety) were discharged after reassurance and were excluded from the study. Children with BCG site reactions due to Kawasaki disease were not included in this study. Statistical analysis was performed using SPSS.

### 2.2 Ethical approval

Ethical approval was obtained from the Joint Research Management Office of Barts Health NHS Trust. Caregiver consent was not required.

## 3 Results

### 3.1 Baseline characteristics

Sixty children presented over a 6 year period at a median age of 6 months (interquartile range (IQR) 4–7 months; range: 2 months–15 years) with BCG vaccine complications. Median age at vaccination was 6 weeks (IQR 2–6 weeks; range: 2 days–15 years) and the male to female ratio was 1.3:1. Forty-one children (68%) were ≤6 months of age at the time of presentation.

### 3.2 Type of complications

Fifty-nine (98%) children presented with local complications and one (2%) with systemic complications. Eighteen children (30%) presented with isolated injection site reactions, 39 (65%) had isolated axillary lymphadenitis (suppurative or non-suppurative) and 2 (3%) had an injection site reaction with lymphadenitis. One child (2%) presented with a BCG site abscess and lymphadenitis and subsequent BCG osteomyelitis, associated with abnormal liver function, indicative of systemic BCG disease.

### 3.3 Management approach

**Fig. 1:**
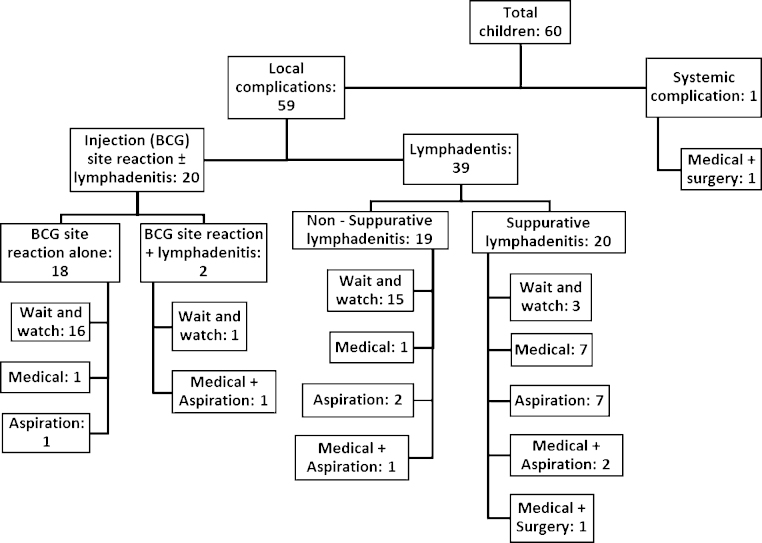
Number of children with BCG vaccine complications and their treatment.

Thirty-five (58%) children were conservatively managed, 15 (25%) received medical therapy and 10 (17%) were managed by aspiration alone (Fig. 1). Of the 15 who received medical therapy, 14 were treated with anti-tuberculous therapy (ATT) and one received co-amoxiclav. Of the 14 children who received ATT, 4 also underwent aspiration and 2 had surgical intervention. Nine children received single ATT (isoniazid) and five children received two-drug ATT (isoniazid and rifampicin). Samples from 16 children were sent for microbiological investigation; BCG was isolated from 8/15 (53%) children with available microbiology results.

### 3.4 Outcomes by clinical presentation

#### 3.4.1 Injection site reaction

**Fig. 2:**
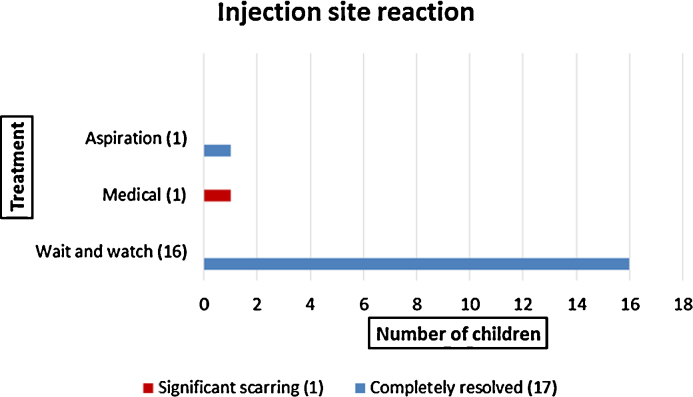
Management and outcome of children with injection site reaction.

Eighteen (30%) children presented with significant injection site reactions. Of these, 10/18 (55%) were less than 6 months of age at the time of presentation. Sixteen children (88%) were managed conservatively, one (6%) received ATT and one (6%) was managed by aspiration alone. Complete resolution was seen in 17/18 (95%) children. One child (5%) was left with persistent scarring at the injection site and was referred to a plastic surgeon for further management (Fig. 2).

#### 3.4.2 Lymphadenitis

**Fig. 3:**
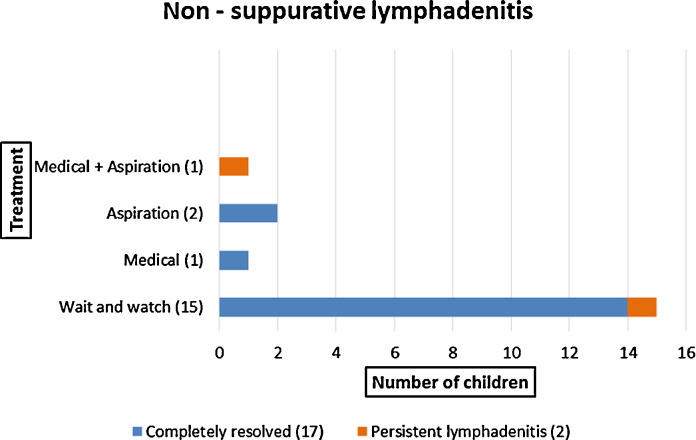
Management and outcome of children with non-suppurative lymphadenitis.

**Fig. 4:**
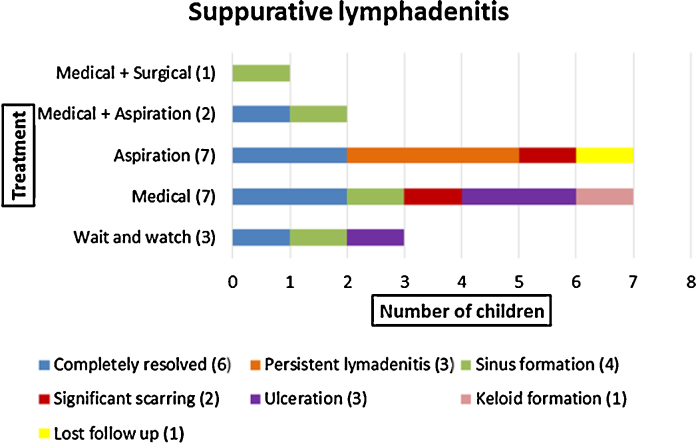
Management and outcome of children with suppurative lymphadenitis.

Thirty-nine (65%) children presented with isolated axillary lymphadenitis (20 with suppurative lymphadenitis; 19 with non-suppurative lymphadenitis). Of these, 28/39 (72%) were less than 6 months of age at the time of presentation. Of those with non-suppurative lymphadenitis, 15/19 (78%) were managed conservatively; 2 (11%) received ATT (one of whom also had aspiration) and 2 (11%) underwent aspiration alone (Fig. 3). Complete resolution was seen in 17/19 (89%) children; two (11%) had persistent lymphadenitis 6 months after presentation. Of those with suppurative lymphadenitis, 3/20 (15%) were treated conservatively, 10/20 (50%) received ATT (of whom 2 had aspiration and 1 had surgical intervention), and 7/20 (35%) underwent aspiration alone (Fig. 4). Complete resolution was seen in only 6/20 (30%) children; 13/20 (65%) had some residual complication and one child (5%) was lost to follow-up after 3 months.

#### 3.4.3 Injection site reaction with lymphadenitis

Two children (3%) presented with significant injection site reaction and lymphadenitis. One child, who had axillary and supraclavicular lymphadenitis together with an injection site reaction, was treated ATT and underwent aspiration; he was left with significant ulceration at the injection site which later resulted in a large persistent scar. He was referred to a plastic surgeon and dermatologist for further management. The second child, who had axillary lymphadenopathy and an injection site reaction, was managed conservatively and recovered completely.

#### 3.4.4 Systemic complications

One child (2%) presented with swelling of the left elbow. He underwent surgery for septic arthritis and was found to have extensive humeral osteomyelitis. Samples collected at operation identified *Mycobacterium tuberculosis* complex by PlexID (Abbott), and BCG was subsequently cultured from the same sample. He was treated with ATT (rifampicin, isoniazid and ethambutol; BCG isolate resistant to pyrazinamide) and later ciprofloxacin was added. Investigations for severe combined immunodeficiency (SCID), human immunodeficiency virus (HIV) and chronic granulomatous disease (CGD) were negative; evaluation of peripheral blood mononuclear cell cytokine production showed normal production of IL-17 and IL-12, but reduced production of IFN-gamma following polyclonal stimuli, suggesting a potential defect in macrophage/T-cell signalling; he subsequently started subcutaneous interferon-gamma therapy.

## 4 Discussion

In this study of children presenting to two large London hospitals with BCG vaccine complications over a 6-year period, approximately one-third presented with injection site complications and two-thirds presented with BCG lymphadenitis; only one child had disseminated BCG disease. Children with injection site complications and non-suppurative lymphadenitis were usually managed conservatively and the vast majority showed complete resolution within 6 months. Among children with suppurative lymphadenitis, there was more variation in management approach: only 15% were managed conservatively, whilst the majority had antituberculous therapy and/or a procedure (aspiration mostly, or surgery); complete resolution was seen in only 30% of cases.

The vast majority of children had local BCG complications and most presented before 6 months of age. Overall, local complications occur in approximately 1:1000 people who receive BCG [4,18], but the risk is higher at younger ages [11,17], likely related to impaired Th1 immunity in early life. Other factors, such as dose and strain of vaccine, administration technique and underlying immunodeficiency or HIV also impact frequency of complications [12,15,18,19]. An injection site abscess can develop up to 30 days after injection [4,16], and was the reason for referral in one-third of cases in our study. A conservative approach is usually adopted for local injection site reaction or abscess [3]. Consistent with this, the vast majority (88%) children referred with isolated injection site reactions in the current study were managed conservatively. The outcome was complete resolution within 6 months in all cases, and a ‘watch and wait’ approach is therefore suitable for these cases.

Management of BCG lymphadenitis, especially suppurative lymphadenitis, still remains unclear and controversial [8,17]. Non-suppurative lymphadenitis may be considered part of the normal course of BCG vaccination, usually resolving spontaneously over a few weeks to months without sequelae [4,8,17,18]. Ipsilateral axillary nodes are most commonly enlarged, but supraclavicular, nuchal and cervical nodes can also be involved and may be considered normal variants [3,13]. Diagnosis is clinical and the absence of any constitutional symptoms is usually sufficient to differentiate suppurative and non-suppurative lymphadenitis. However, non-suppurative lymphadenitis progresses to suppurative lymphadenitis in about 15–30% of cases [8,20], and these nodes can rupture, leading to sinus and fistula formation. Healing in such cases typically takes many months and residual complications may be seen [17,21]. In our study, half of children referred for BCG lymphadenitis had evidence of suppuration and treatment approach varied between conservative management, anti-tuberculous therapy, node aspiration, or combined ATT and aspiration.

The efficacy of anti-tuberculous therapy in treating local complications of BCG is unclear [4,17,22]. Use of ATT is limited by the lack of penetration of drugs into the abscess cavity [4] and inherent resistance of *M. bovis* to pyrazinamide [17,22]. Rifampicin resistance can develop in BCG during treatment [22]. Moreover, routine use of antimicrobials does not reduce the frequency of suppuration in children with non-suppurative lymphadenitis [4,21]. Since abscess-forming lymphadenitis is associated with increased morbidity (such as sinus formation, perforation and ulceration), which can persist for several months, needle aspiration is often advised [4,8,17,20]. In a randomized study from Iran, needle aspiration led to greater regression of lymphadenitis at 2, 4 and 6 months compared to no intervention, and reduced the risk of sinus formation, although some infants needed repeated aspirations [4,23]. A small number (<5%) of children who undergo aspiration may subsequently require surgical intervention [4,23]. In our study, needle aspiration was done in children who either presented with a large abscess at the injection site or a fluctuant node on examination. It was performed by a doctor in the outpatient clinic (predominantly at one hospital only) using local anaesthetic spray and a 21-ga needle. Of the children who underwent aspiration, one developed a sinus, but it is unknown whether this was related to the aspiration procedure.

Although surgical excision is likely to be curative in suppurative lymphadenitis and might reduce the healing time, it requires general anaesthesia, which carries a risk in itself [4,8,17,24]. Surgical excision is not therefore recommended as a routine first-line approach; needle aspiration is considered a safer option [8,23]. Surgery might be needed where aspiration has failed or when the nodes have discharged spontaneously forming a sinus [8,23,24]. The addition of ATT to surgical excision has not been found to be beneficial [4,24]. Only one child with local complications in our study had surgical excision; he presented with recurrent suppurative lymphadenitis despite ATT and three needle aspirations.

Systemic complications of BCG vaccine are extremely rare in developed countries. They can present as osteitis or disseminated BCG disease. The incidence of osteitis/osteomyelitis has been reported as 1–700 per million vaccinated children and depends on the vaccine strain used [12,14,15,25,26]. Prognosis of BCG osteitis/osteomyelitis is generally good without any sequelae [9,10,25,27]. The incidence of disseminated disease is estimated to be about 2 to 3.4 per million vaccinated children [3,9,17,25,28], although one Canadian study [29] reported a rate of 205 per million doses among children from TB-endemic communities. Disseminated disease is associated with case fatality rates of 80–85% [3,4,10,17,30]. Systemic complications usually occur within 6 months of vaccination and are most commonly seen in children with underlying primary immunodeficiency (severe combined immunodeficiency, chronic granulomatous disease, disorders of the gamma-IFN/IL-12 pathway, or a group of conditions termed Mendelian Susceptibility to Mycobacterial Disease (MSMD)) [4,17,19,28–30]. In areas of high HIV prevalence, disseminated BCG disease in the context of undiagnosed infant HIV infection can occur [22,30]. Occasionally, a BCG immune reconstitution inflammatory syndrome (IRIS) can occur in previously vaccinated children after initiation of antiretroviral therapy, presenting as either local site reactions, lymphadenitis or systemic disease [22,31]. Rarely, systemic reactions can be idiopathic [4,19,30,32]. The patient in our study with systemic complication was investigated for possible immunodeficiency and had reduced gamma-IFN production in response to polyclonal stimulation, indicating a potential immunodeficiency, which is currently being better characterized.

This study described all available cases of BCG complications seen over a 6-year period at two large hospitals, but has some limitations. First, this was a hospital-based study, and we are unable to provide an estimate of the overall complication rate associated with BCG vaccination, since the majority of children with mild complications are managed in the community. Second, this was a retrospective study and management approach varied by site and by individual physician practice; we are therefore unable to comment meaningfully on whether the management approach adopted influenced outcome.

In summary, one-third of children referred with BCG complications had injection site reactions; these were mostly managed conservatively and almost all resolved completely, supporting the generally adopted ‘watch and wait’ approach for this type of complication. Two-thirds of children were referred with lymphadenitis following BCG vaccination; in half of these cases, there was evidence of suppuration. There is no clear consensus about the management of suppurative BCG lymphadenitis; the approach taken in our hospitals varied between cases and only one-third of children had complete resolution. Our experience with aspiration is that it is easy to perform in an outpatient setting and is well tolerated by infants and their parents, but whether it reduces the need for surgery or hastens recovery is unknown. Surgery is rarely required and should be reserved for cases where repeat aspirations have failed and definitive node removal is required. Disseminated BCG disease is rare but children should be investigated for underlying immunodeficiency and referred to a specialist centre for management.

## Conflict of interest statement

The authors have no conflicts of interest to declare.
